# Supplementary material for: Comparison of COVID-19 and Non-COVID-19 Tracheostomised Patients: Complications, Survival, and Mortality Risk Factors
Source: J Clin Med. 2025 Jan 19;14(2):633. doi: 10.3390/jcm14020633 (PMC11765842; doi:10.3390/jcm14020633)
Supplement: Supplementary file 1 [file jcm-14-00633-s001.zip › jcm-3409489-supplementary.pdf]

Supplementary Table S1. Baseline characteristics of COVID-19 tracheostomized patients.

	[ALL]	Percutaneous Tracheostomy (PTrach)	Surgical Tracheostomy (STrach)	*p* overall
	N=114	*N=73*	*N=41*	
**Sex:**				0.271
Men	86 (75.4%)	58 (79.5%)	28 (68.3%)	
Women	28 (24.6%)	15 (20.5%)	13 (31.7%)	
**Age:**	61.5 (SD 10.5)	60.9 (SD 11.1)	62.6 (SD 9.58)	0.373
**BMI (kg/m2):**	30.6 (SD 5.48)	30.9 (SD 5.32)	30.0 (SD 5.79)	0.433
BMI categorized (kg/m2):				1.000
Non-obese	57 (51.8%)	36 (51.4%)	21 (52.5%)	
Obesity	47 (42.7%)	30 (42.9%)	17 (42.5%)	
Morbid Obesity	6 (5.45%)	4 (5.71%)	2 (5.00%)	
**Smoking:**				0.371
No	68 (60.7%)	41 (56.9%)	27 (67.5%)	
Yes	44 (39.3%)	31 (43.1%)	13 (32.5%)	
**Sars-CoV2 Vaccination:**				1.000
No	91 (81.2%)	58 (81.7%)	33 (80.5%)	
Yes	21 (18.8%)	13 (18.3%)	8 (19.5%)	
**Comorbidities**				
HTA:				0.583
No	96 (84.2%)	63 (86.3%)	33 (80.5%)	
Yes	18 (15.8%)	10 (13.7%)	8 (19.5%)	
Immunosuppression:				0.521
No	103 (90.4%)	67 (91.8%)	36 (87.8%)	
Yes	11 (9.65%)	6 (8.22%)	5 (12.2%)	
Heart failure:				0.720
No	105 (92.1%)	68 (93.2%)	37 (90.2%)	
Yes	9 (7.89%)	5 (6.85%)	4 (9.76%)	
Autoimmune disease:				0.455
No	106 (93.0%)	69 (94.5%)	37 (90.2%)	
Yes	8 (7.02%)	4 (5.48%)	4 (9.76%)	

Chronic obstructive pulmonary disease (COPD):				1.000
No	113 (99.1%)	72 (98.6%)	41 (100%)	
Yes	1 (0.88%)	1 (1.37%)	0 (0.00%)	
Diabetes mellitus (DM):				0.737
No	97 (85.1%)	61 (83.6%)	36 (87.8%)	
Yes	17 (14.9%)	12 (16.4%)	5 (12.2%)	
Neuromuscular Disease:				0.158
No	109 (95.6%)	68 (93.2%)	41 (100%)	
Yes	5 (4.39%)	5 (6.85%)	0 (0.00%)	
Ischemic cardiopathy (IC):				1.000
No	110 (96.5%)	70 (95.9%)	40 (97.6%)	
Yes	4 (3.51%)	3 (4.11%)	1 (2.44%)	
**Prone position:**				0.535
No	2 (1.75%)	2 (2.74%)	0 (0.00%)	
Yes	112 (98.2%)	71 (97.3%)	41 (100%)	
**Anticoagulant treatment:**				0.709
No	8 (7.02%)	6 (8.22%)	2 (4.88%)	
Yes	106 (93.0%)	67 (91.8%)	39 (95.1%)	
**Ventilatory parameters:**				
PaFi 1st day in ICU (mmHg)	122 (SD 57.3)	115 (SD 50.9)	133 (SD 65.9)	0.146
PEEP 7th day in ICU (PEEP7) (cmH2O)	10.4 (SD2.35)	11.0 (SD 2.17)	9.28 (SD 2.29)	<0.001 *
PaFi 7th day in ICU (PaFi7) (mmHg)	150 (SD 55.0)	147 (SD 53.2)	154 (SD 58.4)	0.528
PEEP pretracheostomy (PEEPt) (cmH2O)	9.26 (SD 11.4)	10.0 (SD 14.1)	7.86 (SD 2.29)	0.203
PaFi pretracheostomy (PaFit) (mmHg)	171 (SD 81.7)	165 (SD 69.9)	180 (SD 98.9)	0.411

**Analytic parameters:**				
D-dimer (µg/L)	4550 (SD 13660)	4283 (SD 14642)	5038 (SD 11818)	0.766
Ferritin (µg/L	8111 (SD 66048)	1493 (SD 1183)	20633 (SD 112231)	0.306
Lactate dehydrogenase (LDH) (U/L)	583 (SD 739)	518 (SD 232)	701 (SD 1203)	0.344
Leukocytes ($x10^9$/L)	1973 (SD 9199)	2462 (SD 10249)	1102 (SD 6983)	0.403
Lymphocytes ($x10^9$/L)	2801 (SD 10867)	3110 (SD 11437)	2246 (SD 9875)	0.676
C-Reactive Protein (CRP) (mg/L)	524 (SD 4175)	126 (SD 126)	1231 (SD 6958)	0.315
International Normalised Ratio (INR)	1.18 (SD 0.35)	1.20 (SD 0.40)	1.15 (SD 0.21)	0.354

*p value statistically significant

Supplementary Table S2. Admission and time intervals studied in tracheostomized COVID-19 (expressed in median and quartiles).

	[ALL]	Percutaneous tracheostomy	Surgical tracheostomy	*p* overall	N
	N=114	*N=73*	*N=41*		
Days from first symptoms to hospital admission	6.00 [4.00;9.00]	6.00 [4.00;8.50]	7.00 [5.00;10.0]	0.201	109
Days from first symptoms to ICU admission	11.0 [8.00;14.0]	11.0 [8.00;14.0]	11.0 [8.00;14.0]	0.850	112
Days of hospital admission	66.5 [44.0;86.8]	65.0 [43.0;83.0]	70.0 [47.0;93.0]	0.469	114
Days of ICU admission	31.0 [5.75;48.0]	31.0 [9.50;48.2]	31.0 [3.50;45.0]	0.566	88
Days of mechanical ventilation	44.0 [33.2;66.8]	42.0 [31.2;50.5]	44.5 [40.5;78.0]	0.135	54
Days of tracheostomy (date tracheostomy to decannulation date)	46.0 [35.0;61.0]	42.0 [35.8;60.5]	47.0 [39.5;76.0]	0.436	53
Days until exitus	15.0 [8.00;39.0]	13.5 [8.75;41.8]	19.0 [8.00;33.0]	0.556	57
Follow-up time	38.5 [12.2;63.0]	38.0 [12.0;58.0]	39.0 [17.0;66.0]	0.839	114

Supplementary Table S3. Complications in tracheostomized COVID-19 patients (Percutaneous vs. Surgical tracheostomy)

	[ALL]	Percutaneous tracheostomy	Surgical tracheostomy	*p* overall
	N=114	*N=73*	*N=41*	
Immediate:				0.757
None	109 (95.6%)	70 (95.9%)	39 (95.1%)	
Bleeding	4 (3.51%)	2 (2.74%)	2 (4.88%)	
Bleeding and ventilation problems	1 (0.88%)	1 (1.37%)	0 (0.00%)	
Postoperative:				0.145
None	98 (86.0%)	64 (87.7%)	34 (82.9%)	
Bleeding	11 (9.65%)	8 (11.0%)	3 (7.32%)	
Ventilation problems	1 (0.88%)	0 (0.00%)	1 (2.44%)	
Bleeding and ventilation problems	4 (3.51%)	1 (1.37%)	3 (7.32%)	

Supplementary Table S4. Discharge circumstances of tracheostomized COVID-19 patients (percutaneous vs. surgical)

	[ALL]	Percutaneous Tracheostomy	Surgical Tracheostomy	*p* overall
	N=114	*N=73*	*N=41*	
Outcomes:				0.909
Home	19 (16.7%)	13 (17.8%)	6 (14.6%)	
Exitus	57 (50.0%)	36 (49.3%)	21 (51.2%)	
Assistance	38 (33.3%)	24 (32.9%)	14 (34.1%)	

Supplementary Table S5. Risk factor of mortality in COVID-19 tracheostomized patients: estimated HR value (HR), standard error (SE), confidence Interval (CI) and p value (p).

	Non-adjusted model				Adjusted Model 1				Adjusted Model 2				Adjusted Model 3				Adjusted Model 4				Adjusted Model 5				Adjusted Model 6			
Predictors	*HR*	*SE*	*CI*	*p*	*HR*	*SE*	*CI*	*p*	*HR*	*SE*	*CI*	*p*	*HR*	*SE*	*CI*	*p*	*HR*	*SE*	*CI*	*p*	*HR*	*SE*	*CI*	*p*	*HR*	*SE*	*CI*	*p*
Surgical Tracheostomy	1.03	0.28	0.60 – 1.77	0.900	1.02	0.30	0.58 – 1.80	0.936	0.97	0.28	0.55 – 1.70	0.918	1.00	0.28	0.57 – 1.73	0.994	1.36	0.43	0.74 – 2.53	0.323	0.88	0.26	0.49 – 1.58	0.666	0.93	0.26	0.53 – 1.62	0.797
Age [every 10 years]					1.02	0.02	1.00 – 1.06	0.104																				
Sex [women]					1.99	0.61	1.09 – 3.61	0.025																				
IMC [Obesity]					1.07	0.31	0.61 – 1.88	0.808																				
IMC [Morbid obesity]					0.67	0.50	0.15 – 2.93	0.589																				
Vaccination [Yes]					0.91	0.32	0.46 – 1.80	0.792																				
Smoking [Yes]					1.81	0.79	0.77 – 4.24	0.171																				
HTA									1.91	0.67	0.96 – 3.78	0.065																
Immunosupression									2.27	1.02	0.93 – 5.50	0.070																
Heart Failure									0.42	0.27	0.12 – 1.45	0.171																
Autoinmune disease									0.32	0.21	0.09 – 1.15	0.080																

Diabetes Mellitus									1.63	0.63	0.76 – 3.48	0.208																
Neuromuscular Disease									0.21	0.21	0.03 – 1.56	0.126																
Ischemic Cardiopaty									5.71	3.73	1.59 – 20.53	0.008																
Prone position									1.32	1.38	0.17 – 10.30	0.791																
Anticoagulant treatment									1.00	0.00	1.00 – 1.00	0.766																
LDH													1.00	0.00	1.00 – 1.00	0.172												
INR													0.78	0.38	0.30 – 2.02	0.611												
PaFi 1st day in ICU (PaFi)																	1.00	0.00	0.99 – 1.01	0.943								
PaFi 7th day ICU admission (PAFI7)																	1.00	0.00	0.99 – 1.00	0.572								
PEEP 7th day ICU admission (PEEP7)																	0.92	0.07	0.79-1.06	0.250								
PEEP pretracheostomy (PEEPt)																	1.06	0.03	1.01 – 1.11	0.017								
PaFi pretracheostomy (PaFit)																	1.00	0.00	0.99 – 1.00	0.062								

INR pretracheostomy (INRt)																	1.04	0.01	1.01 – 1.07	0.004								
D-dimer																					1.00	0.00	1.00 – 1.00	0.863				
Ferritin																					1.00	0.00	1.00 – 1.00	0.343				
Leukocytes																					1.00	0.00	1.00 – 1.00	0.801				
Lymphocytes																					1.00	0.00	1.00 – 1.00	0.749				
Surgical Complications [Bleeding]																									0.32	0.32	0.04 – 2.32	0.259
Surgical Complications [Bleeding + Ventilation problems]																									0.00	0.00	0.00 – Inf	0.996
Postoperative complications [Bleeding]																									0.77	0.36	0.30 – 1.94	0.574
Postoperative complications [Ventilation problems]																									8.63	9.11	1.09 – 68.26	0.041
Postoperative complications [Bleeding+Ventilation problems]																									1.33	0.81	0.40 – 4.37	0.642

Supplementary Table S6. cohort baseline characteristics (Non-COVID-19 Historical tracheostomized patients).

	[ALL]	HPTrach	HSTrach
	N=231	N=67	N=164
Sex:			
Men	161 (69.7%)	47 (70.1%)	114 (69.5%)
Women	70 (30.3%)	20 (29.9%)	50 (30.5%)
Age:	59.4 (15.3)	60.0 (15.6)	59.1 (15.2)
	62.0 [51.0;70.0]	62.0 [53.5;70.5]	62.0 [49.8;70.0]
Obesity:			
No	224 (97.0%)	66 (98.5%)	158 (96.3%)
Yes	7 (3.03%)	1 (1.49%)	6 (3.66%)
Diabetes Mellitus:			
No	206 (89.2%)	61 (91.0%)	145 (88.4%)
Yes	25 (10.8%)	6 (8.96%)	19 (11.6%)
HTA:			
No	166 (71.9%)	51 (76.1%)	115 (70.1%)
Yes	65 (28.1%)	16 (23.9%)	49 (29.9%)
COPD:			
No	219 (94.8%)	65 (97.0%)	154 (93.9%)
Yes	12 (5.19%)	2 (2.99%)	10 (6.10%)
Heart Failure:			
No	212 (91.8%)	65 (97.0%)	147 (89.6%)
Yes	19 (8.23%)	2 (2.99%)	17 (10.4%)
Smoking:			
No	212 (91.8%)	63 (94.0%)	149 (90.9%)
Yes	19 (8.23%)	4 (5.97%)	15 (9.15%)

Supplementary Table S7. Tracheostomy complications of the Historical cohort (Non-COVID-19 tracheostomized patients)

	[ALL]	HPTrach	HSTrach	p.overall
	N=200	N=67	N=133	
Bleeding:				0.659
No	181 (90.5%)	62 (92.5%)	119 (89.5%)	
Yes	19 (9.50%)	5 (7.46%)	14 (10.5%)	
Ventilatory:				1.000
No	186 (93.0%)	62 (92.5%)	124 (93.2%)	
Yes	14 (7.00%)	5 (7.46%)	9 (6.77%)	
Stenosis:				0.721
No	191 (95.5%)	65 (97.0%)	126 (94.7%)	
Yes	9 (4.50%)	2 (2.99%)	7 (5.26%)	

Supplementary Table S8. Discharge circumstances of Historical cohort patients

	[ALL]	HPTrach	HSTrach	p.overall
	N=231	N=67	N=164	
Outcomes:				0.279
Home	27 (11.7%)	8 (11.9%)	19 (11.6%)	
Exitus	63 (27.3%)	23 (34.3%)	40 (24.4%)	
Assistance	141 (61.0%)	36 (53.7%)	105 (64.0%)	

Supplementary Table S9: Mortality incidence in tracheostomized patients

**Sample type**	**N**	**n**	**% (CI95%)**	**Patients-Years**	**Rate for 1000 patients-year**
Non-COVID-19	200	58	29 (22.82, 35.82)	8845	6.56 (4.98, 8.41)
HPTrach	67	23	34.33 (23.15, 46.94)	2519	9.13 (5.79; 13.46)
HSTrach	133	35	26.32 (19.06, 34.65)	6326	5.53 (3.85, 7.6)
COVID-19	114	57	50 (40.49, 59.51)	4498	12.67 (9.6, 16.29)
PTrach	73	36	49.32 (37.4, 61.28)	2842	12.67 (8.87, 17.33)
STrach	41	21	51.22 (35.13, 67.12)	1656	12.68 (7.85, 19.02)

Supplementary Table S10. Mortality risk factors in Non-COVID-19 tracheostomized patients: estimated HR value (HR), standard error (SE), confidence Interval (CI) and p value (p)

	Non-adjusted model				Adjusted model 1				Adjusted model 2			
Predictors	*HR*	*SE*	*CI*	*p*	*HR*	*SE*	*CI*	*p*	*HR*	*SE*	*CI*	*p*
Surgical Tracheostomy	0.40	0.12	0.22 – 0.71	0.002	0.39	0.12	0.22 – 0.71	0.002	0.33	0.10	0.17 – 0.61	<0.001
Age [10 years]					1.02	0.01	1.00 – 1.04	0.104	1.02	0.01	0.99 – 1.04	0.152
Sex [women]					1.43	0.44	0.79 – 2.60	0.236	1.64	0.53	0.87 – 3.07	0.124
Obesity									0.55	0.57	0.07 – 4.22	0.563
Diabetes Mellitus									0.87	0.42	0.33 – 2.26	0.771
HTA									1.04	0.37	0.52 – 2.09	0.904
COPD									2.13	1.34	0.62 – 7.28	0.227
Heart failure									3.85	1.70	1.62 – 9.17	0.002

Smoking									2.31	1.13	0.89 – 6.00	0.087

Supplementary Table S11: Discharge circumstances and complications of COVID-19 vs. Non COVID-19 Tracheostomized patients

	[ALL]	Non-COVID-19 tracheostomized patients (Historical cohort)	COVID-19 tracheostomized patients	p overall
	N=345	N=231	N=114	
Outcomes:				<0.001
Home	46 (13.3%)	27 (11.7%)	19 (16.7%)	
Exitus	120 (34.8%)	63 (27.3%)	57 (50.0%)	
Assistance	179 (51.9%)	141 (61.0%)	38 (33.3%)	
Complications:				1.000
No	255 (81.2%)	162 (81.0%)	93 (81.6%)	
Yes	59 (18.8%)	38 (19.0%)	21 (18.4%)	

Supplementary Table S12. Mortality risk factors for tracheostomized patients (COVID-19 and non-COVID-19): estimated HR value (HR), standard error (SE), confidence Interval (CI) and p value (p)

	Non-adjusted Model				Adjusted Model 1				Adjusted Model 2			
Predictors	*HR*	*SE*	*CI*	*p*	*HR*	*SE*	*CI*	*p*	*HR*	*SE*	*CI*	*p*
Surgical tracheostomy	0.66	0.14	0.43 – 1.00	0.049								
COVID19 [yes]	2.09	0.44	1.38 – 3.17	0.001								
Age [10 years]					1.22	0.10	1.03 – 1.44	0.021	1.21	0.11	1.01 – 1.44	0.034
Sex [women]					1.76	0.37	1.16 – 2.67	0.008	1.55	0.33	1.01 – 2.36	0.044

Percutaneous Tracheostomy and COVID19					0.70	0.21	0.38 – 1.26	0.230	0.57	0.19	0.30 – 1.09	0.089
Surgical tracheostomy and COVID19					0.28	0.08	0.15 – 0.50	<0.001	0.18	0.06	0.09 – 0.35	<0.001
Obesity									0.91	0.24	0.54 – 1.54	0.730
Diabetes Mellitus									1.25	0.37	0.71 – 2.22	0.441
HTA									1.49	0.38	0.90 – 2.46	0.122
COPD									1.99	1.06	0.70 – 5.66	0.196
Heart failure									3.35	1.19	1.66 – 6.74	0.001
Smoking									2.28	0.73	1.22 – 4.26	0.009
Observations	314				314				310			
